# Supplementary material for: Analysis of PPARγ Signaling Activity in Psoriasis
Source: Int J Mol Sci. 2021 Aug 10;22(16):8603. doi: 10.3390/ijms22168603 (PMC8395241; doi:10.3390/ijms22168603)
Supplement: Supplementary file 1 [file ijms-22-08603-s001.zip › Supplemental materials_Analysis of PPARg signaling activity in psoriasis/Pathway models/Models images and html files/Differentiation of psoriatic T cells/6174.html]

IFNA1


# Protein IFNA1

|  |  |
| --- | --- |
| URN | urn:agi-llid:3439 |
| Total Entities | 0 |
| Connectivity | 1239 |
| Name | IFNA1 |
| Description | interferon, alpha 1 |
| Notes | The protein encoded by this gene is produced by macrophages and has antiviral activity. This gene is intronless and the encoded protein is secreted. [provided by RefSeq, Sep 2011] |
| Primary Cell Localization | Extracellular |
| Class | Ligand |
| ObjectType | Protein |

---

|  |  |
| --- | --- |
| Pathway | Cortisol in Resolving Inflammation |
|  | Toll-like Receptors Act through MYD88-TIRAP Signaling |
|  | Antiviral Signaling through Pattern Recognition Receptors |
|  | Toll-like Receptors Act through MYD88 Signaling |
|  | TLR4 Signaling in Leukocytes |
|  | Toll-like Receptors in Sterile Inflammation |
|  | AXL Receptor Inhibits Macrophages and Dendritic Cells Function |
|  | Dendritic Cells Function in Atherosclerosis |
|  | MAVS in Antiviral Innate Immune Response in Myocarditis |
|  | Proteins Involved in Myocarditis |
|  | SOCS in Negative Feedback Regulation in Myocarditis |
|  | Toll-like Receptors (TLR) in Antiviral Innate Immune Response |
|  | T-Cells Differentiation Block in Psoriasis |
|  | Dendritic Cells Function in Psoriasis |
|  | Dendritic Cell Activation in Systemic Lupus Erythematosis |
|  | Proteins Involved in Systemic Lupus Erythematosus |
|  | Th17-Cell Function in Systemic Lupus Erythematosus |
|  | B-Cell Activation in Systemic Lupus Erythematosus |
|  | Neutrophil and Macrophage Function in Systemic Lupus Erythematosus |
|  | Proteins Involved in Vitiligo |
|  | Natural Killer T-Cell Roles in Diabetes Mellitus Type 1 |
|  | Plasmacytoid Dendritic Cell in Diabetes Mellitus Type 1 |
|  | TLR7 and TLR9 in Plasmacytoid Dendritic Cell |
|  | Hashimoto's Thyroiditis Triggers |
|  | Dendritic Cell Function in Ulcerative Colitis |
|  | Dendritic Cell Dysfunction in Psoriatic Arthritis |
|  | Toll-like Receptors (TLR) in Periodontitis |
|  | Alveolar Macrophages Dysfunction in COPD |
|  | IFNA1/IFNR Expression Targets |
|  | IFNG/IFNR Expression Targets |
|  | IL2 Expression Targets |
|  | IFNA1/Gq Expression Targets |
|  | CD40LG -> NF-kB/ELK/SRF -> CREB/NFATC Expression Targets |
|  | TLR3 Expression Targets |
|  | pathway 4 |
|  | PPAR Psoriasis |
|  | New Pathway (3) |
|  | dsRNA induces innate immunity and INFs in alveolar cells |
|  | IFN signaling in alveolar cells |
|  | Innate immunity in Leukocytes |
|  | AGER feed forward loop in innate immunity |
|  | Dendritic Cell Activation in COVID19 |
|  | 1\_Differentiation of psoriatic T cells |
|  | 1\_Insufficient activation of immune response in the middle ear epithelium cells in otitis media |
|  | 2\_Intestine epithelial cells drive immunological responses in ulcerative colitis |
|  | 1\_Antigen-presenting cells promote islet dysfunction and immune system activation in T1D |
|  | Differentiation of psoriatic T cells |

---

|  |  |
| --- | --- |
| MedScan ID | 3439 |
|  | 602857 |
|  | 654874 |

---

|  |  |
| --- | --- |
| LocusLink ID | 3439 |
|  | 100365568 |
|  | 15970 |
|  | 502959 |
|  | 242519 |
|  | 242517 |
|  | 678807 |
|  | 15969 |
|  | 230398 |
|  | 638105 |
|  | 230396 |
|  | 15964 |
|  | 15974 |
|  | 690894 |
|  | 15962 |
|  | 404549 |
|  | 15972 |
|  | 15968 |
|  | 680208 |
|  | 15965 |

---

|  |  |
| --- | --- |
| Alias | IFN I |
|  | interferon alpha 1 |
|  | alpha interferon 8/6 |
|  | alpha interferon 11 |
|  | RP23-67C1.6 |
|  | alpha interferon 1b |
|  | OTTMUSP00000008117 |
|  | Ifa9 |
|  | RP23-139P14.1 |
|  | ifn-alpha 1 |
|  | alpha 12 interferon |
|  | MGC129421 |
|  | MGC138505 |
|  | alpha 1b interferon |
|  | OTTMUSP00000012086 |
|  | alpha 6-like interferon |
|  | OTTMUSP00000012104 |
|  | RP23-360M23.1 |
|  | RP23-139P14.26 |
|  | MGC143604 |
|  | alpha interferon 12 |
|  | alpha interferon 9 |
|  | ifn-alpha 1b |
|  | IFNA1 |
|  | alpha 1 interferon |
|  | interferon alpha 1b |
|  | RP23-67C1.1 |
|  | IFN A1 |
|  | OTTMUSP00000008112 |
|  | RP23-139P14.29-001 |
|  | OTTMUSP00000012096 |
|  | OTTHUMP00000045110 |
|  | MGC155959 |
|  | IFN-(a)11 |
|  | alpha interferon 1 |
|  | RP23-139P14.26-001 |
|  | IFN1 |
|  | OTTMUSP00000008118 |
|  | MGC138507 |
|  | OTTMUSP00000008138 |
|  | RP23-139P14.27-001 |
|  | MGC129420 |
|  | ifn-alpha 8/6 |
|  | alpha 11 interferon |
|  | RP23-67C1.2 |
|  | OTTMUSP00000012102 |
|  | RP23-139P14.29 |
|  | alpha interferon 6-like |
|  | LOC89955 |
|  | INA I |
|  | alpha 8/6 interferon |
|  | Ifa1 |
|  | INA1 |
|  | OTTMUSP00000008120 |
|  | alpha 9 interferon |
|  | IFNA I |
|  | Ifa I |
|  | MGC138207 |
|  | RP23-67C1.4 |
|  | IFL |
|  | IFN |
|  | IFNA@ |
|  | IFNA13 |
|  | IFN-ALPHA |
|  | IFN-alphaD |
|  | IFN-alpha-1/13 |
|  | interferon alpha-D |
|  | interferon-alpha1 |
|  | leIF D |
|  | interferon alpha-1/13 |
|  | LOC384101 |
|  | LOC384100 |
|  | OTTMUSG00000007655 |
|  | IFN alpha 12 |
|  | RP23-139P14.3 |
|  | LOC638105 |
|  | LOC680208 |
|  | IFN-alpha-8 |
|  | IFN-alpha-7 |
|  | interferon alpha-2 |
|  | interferon alpha-1 |
|  | IFN-alpha-9 |
|  | interferon alpha-8 |
|  | interferon alpha-7 |
|  | interferon alpha-6 |
|  | IFN-alpha-6 |
|  | IFN-alpha-5 |
|  | interferon alpha-5 |
|  | RP23-139P14.5 |
|  | interferon alpha 12 |
|  | RP23-139P14.4 |
|  | interferon alpha 11 |
|  | IFN-alpha-2 |
|  | IFN-alpha-1 |
|  | OTTMUSG00000007662 |
|  | interferon alpha family, gene 2 |
|  | interferon alpha family, gene 5 |
|  | interferon alpha family, gene 6 |
|  | IFN-[a]11 |
|  | interferon alpha family, gene 9 |
|  | interferon alpha family, gene 7 |
|  | interferon alpha 8/6 precursor; IFNa8/6 |
|  | interferon alpha family, gene 8 |
|  | interferon alpha family, gene B |
|  | LOC678807 |
|  | interferon alpha 6-like |
|  | LOC100365568 |
|  | limitin |
|  | interferon alpha-9 |
|  | alpha-interferon |
|  | ifn-alpha 11 |
|  | ifn-alpha 12 |
|  | IFN-alpha-11 |
|  | IFN-alpha-13 |
|  | IFN-alpha-12 |
|  | RGD1561246 |
|  | Ifnaa |
|  | interferon alpha 9 |
|  | interferon alpha-7-like |
|  | interferon, alpha 14 |
|  | Ifnat6 |
|  | OTTMUSG00000011275 |
|  | similar to put. precursor MuIFN-alpha 5 |
|  | Gm13280 |
|  | Gm12597 |
|  | LOC230403 |
|  | LOC329879 |
|  | Ifna11 |
|  | Ifna12 |
|  | Ifna8 |
|  | interferon alpha family gene 1 |
|  | Ifna9 |
|  | similar to alpha-interferon |
|  | Ifa2 |
|  | interferon alpha-11 |
|  | BC114392 |
|  | interferon alpha-13 |
|  | interferon alpha-12 |
|  | Ifa6 |
|  | Ifa7 |
|  | Ifa5 |
|  | interferon alpha family, gene 13 |
|  | Ifa8 |
|  | interferon alpha family, gene 11 |
|  | interferon alpha 6T |
|  | novel interferon alpha (Ifna) family member |
|  | LOC384099 |
|  | Ifna6T |
|  | ifn-alpha 9 |
|  | LOC298209 |
|  | MuIFN-alpha-A |
|  | interferon alpha 8/6 |
|  | IFNa8/6 |
|  | OTTMUSP00000012094 |
|  | OTTMUSP00000012097 |
|  | OTTMUSP00000012091 |
|  | OTTMUSP00000012095 |
|  | Ifna5 |
|  | Mrpl48-ps |
|  | IFN-alpha-B |

---

|  |  |
| --- | --- |
| GO ID | 0005125 |
|  | 0005132 |
|  | 0030183 |
|  | 0042100 |
|  | 0002286 |
|  | 0002250 |
|  | 0007596 |
|  | 0019221 |
|  | 0051607 |
|  | 0006959 |
|  | 0002323 |
|  | 0033141 |
|  | 0043330 |
|  | 0060337 |
|  | 0005576 |
|  | 0005615 |
|  | 0010469 |
|  | 0005126 |
|  | 0006952 |
|  | 0009615 |
|  | 0045087 |
|  | 0045343 |
|  | 0060338 |
|  | 0042742 |

---

|  |  |
| --- | --- |
| KEGG ID | hsa:3439 |
|  | hsa:3447 |
|  | mmu:15970 |
|  | rno:680208 |
|  | rno:690894 |
|  | mmu:15972 |
|  | mmu:15962 |
|  | mmu:15974 |
|  | mmu:15964 |
|  | mmu:15965 |
|  | mmu:242517 |
|  | mmu:15968 |
|  | mmu:404549 |
|  | mmu:242519 |
|  | mmu:15969 |
|  | mmu:230396 |
|  | rno:502959 |
|  | mmu:230398 |

---

|  |  |
| --- | --- |
| Organism | Homo sapiens {Organism urn:agi-taxid:9606} |
|  | Homo sapiens |
|  | Mus musculus |
|  | Rattus norvegicus |

---

|  |  |
| --- | --- |
| Mouse chromosome position | 4 42.01 cM |
|  | 4 |
|  | 4 42.6 cM |
|  | 4 42.04 cM |
|  | 4 42.03 cM |
|  | 4 42.02 cM |

---

|  |  |
| --- | --- |
| OMIM ID | 147660 |

---

|  |  |
| --- | --- |
| Rat chromosome position | 5q32 |
|  | 5 |
|  | 3 |

---

|  |  |
| --- | --- |
| Hugo ID | 5417 |
|  | 5419 |
|  | HGNC:5417 |

---

|  |  |
| --- | --- |
| Human chromosome position | 9p21.3 |
|  | 9p22 |

---

|  |  |
| --- | --- |
| Swiss-Prot Accession | L0N195 |
|  | P01562 |
|  | P01562.1 |
|  | D4Q9M8 |
|  | Q14605 |
|  | Q2M1L8 |
|  | Q52LB8 |
|  | Q5VYQ2 |
|  | Q7M4Q1 |
|  | Q8WZ68 |
|  | Q9UMJ3 |
|  | P07349 |
|  | P06799 |
|  | Q80SS5 |
|  | Q80SU4 |
|  | Q810G5 |
|  | Q810G6 |
|  | B1AWY7 |
|  | Q810G7 |
|  | Q810G1 |
|  | Q810G2 |
|  | Q810G3 |
|  | P07350 |
|  | Q810G4 |
|  | Q61716 |
|  | P09235 |
|  | Q61718 |
|  | Q61719 |
|  | P01573 |
|  | P01572 |
|  | B1AYH8 |
|  | B1AYH7 |
|  | Q7M0A3 |
|  | Q1WWM6 |
|  | Q496V3 |
|  | Q496W3 |
|  | Q149I5 |
|  | Q0IJ79 |
|  | Q6XL62 |
|  | Q56VR2 |
|  | Q1WWN2 |
|  | D3YU14 |

---

|  |  |
| --- | --- |
| PIR ID | C23285 |
|  | F42753 |
|  | H42753 |
|  | S62682 |
|  | A24401 |
|  | A01836 |
|  | I49775 |
|  | I49774 |

---

|  |  |
| --- | --- |
| GenBank ID | NC\_000009 |
|  | NM\_024013 |
|  | NP\_076918 |
|  | AB445100 |
|  | BAI99735 |
|  | AF439447 |
|  | AAL35223 |
|  | AL353732 |
|  | CH471071 |
|  | EAW58609 |
|  | CQ847555 |
|  | CAH18030 |
|  | DQ185447 |
|  | ABA03167 |
|  | GM863573 |
|  | CAT16481 |
|  | J00210 |
|  | AAB59403 |
|  | M29884 |
|  | AAA52714 |
|  | AB578886 |
|  | BAM72353 |
|  | BC074928 |
|  | AAH74928 |
|  | BC074929 |
|  | AAH74929 |
|  | BC112300 |
|  | AAI12301 |
|  | BC112302 |
|  | AAI12303 |
|  | E00175 |
|  | JN848523 |
|  | AET86952 |
|  | V00537 |
|  | CAA23798 |
|  | V00538 |
|  | CAA23799 |
|  | P01562 |
|  | AAI00700 |
|  | BC116870 |
|  | BC116872 |
|  | NP\_996858 |
|  | AAI25322 |
|  | AAI40286 |
|  | AAI60330 |
|  | AAI30232 |
|  | AAI12003 |
|  | AAI14424 |
|  | BC116864 |
|  | L38698 |
|  | BC116862 |
|  | XM\_001053102 |
|  | M68944 |
|  | HQ258738 |
|  | AAO38687 |
|  | BC160330 |
|  | AAO38688 |
|  | ADR83489 |
|  | ABBA01065176 |
|  | BC130231 |
|  | AY220461 |
|  | CAM14915 |
|  | AY220462 |
|  | P09235 |
|  | AY220464 |
|  | AY220465 |
|  | CAM14919 |
|  | CAM14918 |
|  | CAM14917 |
|  | AC\_000026 |
|  | NP\_996750 |
|  | NP\_996753 |
|  | NP\_996754 |
|  | BC140285 |
|  | NC\_000070 |
|  | EDL30976 |
|  | AAHY01037858 |
|  | AAHY01037857 |
|  | EDL30974 |
|  | EDL30975 |
|  | AAHY01037859 |
|  | EDL30972 |
|  | EDL30970 |
|  | AMYH02019286 |
|  | AAHY01037851 |
|  | AAHY01037854 |
|  | AAHY01037853 |
|  | AAHY01037855 |
|  | AAI20911 |
|  | AABR06038503 |
|  | AL772394 |
|  | AAI20912 |
|  | M23840 |
|  | XP\_001053102 |
|  | AAA37889 |
|  | AL928605 |
|  | AY266684 |
|  | AAI00699 |
|  | EDL30961 |
|  | AAA37885 |
|  | AAA37886 |
|  | NC\_018920 |
|  | EDL30960 |
|  | AAA37888 |
|  | AAI00690 |
|  | NM\_008333 |
|  | NM\_008336 |
|  | NM\_008334 |
|  | BC125321 |
|  | AAA37883 |
|  | EDL30969 |
|  | AAI51088 |
|  | EDL30968 |
|  | P06799 |
|  | AAI00689 |
|  | BC141454 |
|  | BC120750 |
|  | AY225950 |
|  | BC114423 |
|  | AAHY01037870 |
|  | BC112002 |
|  | AY225953 |
|  | AY225954 |
|  | AY225951 |
|  | AY225952 |
|  | EDL30958 |
|  | P01573 |
|  | P01572 |
|  | AAHY01037869 |
|  | BC141569 |
|  | D00460 |
|  | AAHY01037867 |
|  | AAI04353 |
|  | AAI04354 |
|  | AAI16217 |
|  | AAP79990 |
|  | AC\_000073 |
|  | K01411 |
|  | AAI14393 |
|  | BC100689 |
|  | P07349 |
|  | BC100688 |
|  | CAM26603 |
|  | NP\_796321 |
|  | CAM26601 |
|  | AAO64455 |
|  | AAO64454 |
|  | AAO64457 |
|  | AAO64458 |
|  | AAI41651 |
|  | Q80SU4 |
|  | BX530016 |
|  | XP\_003946183 |
|  | XP\_003754100 |
|  | P07350 |
|  | CH466527 |
|  | AAI17776 |
|  | Q61716 |
|  | AAI17775 |
|  | X01969 |
|  | AAI48714 |
|  | M13710 |
|  | NP\_032362 |
|  | NP\_032360 |
|  | NP\_032359 |
|  | AAI20725 |
|  | BC116216 |
|  | AAI20723 |
|  | X01974 |
|  | X01971 |
|  | X01972 |
|  | XM\_001056120 |
|  | BC141650 |
|  | CAM22267 |
|  | Q80SS5 |
|  | XM\_003946134 |
|  | NP\_796335 |
|  | BC100699 |
|  | BC100698 |
|  | XP\_578464 |
|  | BC148713 |
|  | XM\_001076062 |
|  | BC120724 |
|  | BC120722 |
|  | NP\_034637 |
|  | NP\_034635 |
|  | NP\_034633 |
|  | NP\_034632 |
|  | NM\_010507 |
|  | XP\_001056120 |
|  | NM\_010505 |
|  | BAA00349 |
|  | NM\_010502 |
|  | NM\_010503 |
|  | M28587 |
|  | CAA26002 |
|  | CAA26003 |
|  | CAA26004 |
|  | AAI20751 |
|  | CAA26006 |
|  | AAI41455 |
|  | NM\_206975 |
|  | AAI41570 |
|  | CAH18031 |
|  | XP\_001076062 |
|  | AC\_000141 |
|  | CAM14939 |
|  | CAM26599 |
|  | CAM26598 |
|  | XM\_578464 |
|  | CQ847557 |
|  | CAH72911 |
|  | CAM14940 |
|  | NC\_005104 |
|  | CAM14942 |
|  | CAM14941 |
|  | AAI16873 |
|  | XP\_006543925 |
|  | AAI16871 |
|  | XM\_003754052 |
|  | NM\_177347 |
|  | BC120911 |
|  | BC120910 |
|  | NM\_206870 |
|  | M13660 |
|  | BC114392 |
|  | NM\_206871 |
|  | BC104353 |
|  | NM\_206867 |
|  | BC104352 |
|  | AAI16865 |
|  | AAI16863 |
|  | AAA58254 |
|  | AAHX01038183 |
|  | AAHX01038185 |
|  | AAO63593 |
|  | AAO63592 |
|  | AAO63595 |
|  | AAO63594 |
|  | BC117774 |
|  | AAO63596 |
|  | BC117775 |
|  | AY190046 |
|  | BC151087 |
|  | AY190047 |
|  | AAR00556 |
|  | XM\_006543862 |
|  | AY226993 |
|  | NM\_177361 |
|  | NT\_008413 |
|  | NW\_001839149 |
|  | NW\_924062 |
|  | AC\_000052 |
|  | NT\_039260 |
|  | NW\_001030747 |
|  | Q810G4 |
|  | Q1WWM6 |
|  | Q496V3 |
|  | Q496W3 |
|  | Q810G2 |
|  | Q149I5 |
|  | Q810G5 |
|  | Q810G6 |
|  | Q0IJ79 |
|  | Q6XL62 |
|  | Q61719 |
|  | Q56VR2 |
|  | Q1WWN2 |
|  | Q810G1 |
|  | Q61718 |
|  | Q810G3 |
|  | NW\_001084841 |
|  | NW\_047715 |
|  | AABR03043885 |
|  | AABR03041340 |
|  | NW\_047673 |

---

|  |  |
| --- | --- |
| Swiss-Prot ID | IFNA1\_HUMAN |
|  | IFNAD\_MOUSE |
|  | IFNA1\_MOUSE |
|  | IFNAC\_MOUSE |
|  | IFNA9\_MOUSE |
|  | Q810G3\_MOUSE |
|  | Q810G5\_MOUSE |
|  | Q810G4\_MOUSE |
|  | Q810G6\_MOUSE |
|  | Q1WWN2\_MOUSE |
|  | Q810G1\_MOUSE |
|  | Q810G2\_MOUSE |
|  | B1AYH8\_MOUSE |
|  | B1AWY7\_MOUSE |
|  | Q61719\_MOUSE |
|  | B1AYH7\_MOUSE |
|  | Q61718\_MOUSE |
|  | Q810G7\_MOUSE |

---

|  |  |
| --- | --- |
| Cell Localization | Secreted |
|  | Extracellular |

---

|  |  |
| --- | --- |
| Ensembl ID | ENSG00000197919 |
|  | ENSP00000276927.1 |
|  | ENST00000276927.2 |
|  | ENSMUSP00000099873 |
|  | ENSMUSP00000099872 |
|  | ENSMUSP00000099871 |
|  | ENSMUSP00000099870 |
|  | ENSMUSP00000092580 |
|  | ENSMUST00000072734 |
|  | ENSMUSG00000096011 |
|  | ENSMUST00000170428 |
|  | ENSMUST00000102809 |
|  | ENSMUSP00000127921 |
|  | ENSMUST00000102808 |
|  | ENSMUST00000102807 |
|  | ENSMUSP00000072517 |
|  | ENSMUST00000102806 |
|  | ENSMUST00000102804 |
|  | ENSMUSG00000073811 |
|  | ENSMUSG00000078353 |
|  | ENSMUSG00000070906 |
|  | ENSMUSG00000070907 |
|  | ENSMUSG00000096682 |
|  | ENSMUSG00000095270 |
|  | ENSMUST00000094972 |
|  | ENSMUSP00000099868 |
|  | ENSP00000276927 |
|  | ENSMUSP00000100777 |
|  | ENSMUSP00000100774 |
|  | ENSMUSG00000095498 |
|  | ENSMUSG00000063376 |
|  | ENSMUSG00000095896 |
|  | ENSMUST00000105143 |
|  | ENSMUST00000105146 |
|  | ENST00000276927 |

---

|  |  |
| --- | --- |
| MGI ID | 107663 |
|  | 3641425 |
|  | 107662 |
|  | 107661 |
|  | 1097683 |
|  | 2667155 |
|  | 107666 |
|  | 3649418 |
|  | 109210 |
|  | 2676324 |
|  | 107659 |
|  | 107668 |
|  | 3649260 |

---

|  |  |
| --- | --- |
| RGD ID | 1561246 |
|  | 1594223 |
|  | 1306135 |

---

|  |  |
| --- | --- |
| Unigene ID | Hs.37026 |
|  | Mm.57127 |
|  | Mm.246618 |
|  | Mm.377947 |
|  | Mm.377089 |
|  | Mm.377246 |
|  | Mm.246592 |
|  | Mm.377261 |
|  | Mm.377087 |
|  | Mm.377092 |
|  | Mm.377093 |
|  | Mm.14091 |
|  | Mm.377090 |
|  | Mm.377091 |

---

|  |  |
| --- | --- |
| Shape | Rhomb |

---

|  |  |
| --- | --- |
| IPI ID | IPI00136875 |
|  | IPI00003072 |
|  | IPI00874708 |
|  | IPI00228625 |
|  | IPI00875452 |
|  | IPI00282746 |
|  | IPI00875058 |
|  | IPI00626044 |
|  | IPI00329860 |
|  | IPI00329856 |
|  | IPI00874594 |
|  | IPI00124768 |
|  | IPI00831249 |
|  | IPI00357663 |
|  | IPI00604550 |
|  | IPI00128158 |
|  | IPI00313852 |
|  | IPI00127803 |
|  | IPI00882013 |
|  | IPI00228642 |
|  | IPI00876513 |
|  | IPI00876157 |
|  | IPI00874487 |
|  | IPI00875342 |
|  | IPI00875168 |
|  | IPI00875365 |
|  | IPI00329859 |
|  | IPI00875571 |
|  | IPI00808187 |
|  | IPI00567677 |
|  | IPI00560574 |
|  | IPI00562295 |
|  | IPI00564392 |

---
